# Evaluation of Sperm Chromatin Integrity Using Aniline Blue and Toluidine Blue Staining in Infertile and Normozoospermic Men

**Published:** 2019

**Authors:** Soheila Pourmasumi, Arezoo Khoradmehr, Tahereh Rahiminia, Parvin Sabeti, Ali Reza Talebi, Jalal Ghasemzadeh

**Affiliations:** 1- Non-Communicable Diseases Research Center, Rafsanjan University of Medical Sciences, Rafsanjan, Iran; 2- Research and Clinical Center for Infertility, Yazd Reproductive Sciences Institute, Shahid Sadoughi University of Medical Sciences, Yazd, Iran; 3- Gametogenesis Research Center, Fertility, and Infertility Center, Kashan University of Medical Sciences, Kashan, Iran.; 4- Department of Anatomy, Faculty of Medicine, Kurdistan University of Medical Sciences, Sanandaj, Iran

**Keywords:** Aniline blue, Infertility, Sperm, Toluidine blue

## Abstract

**Background:**

Male infertility is defined as a man lost his ability to fertilize a fertile female naturally. Diagnosis of male infertility cannot be made just according to basic semen analysis. It is necessity to have specific tests for evaluation of chromatin integrity. In this study, an attempt was made to evaluate the sperm chromatin quality in fertile men and infertile subgroup.

**Methods:**

Among 1386 couples, 342 men were categorized into normospermia and 1044 were infertile and they were referred to Yazd Research and Clinical Center for infertility treatment. Standard semen analysis and sperm nuclear maturity tests including aniline blue (AB) and toluidine blue (TB) staining were done. Data were analyzed by SPSS software. The p≤0.05 was considered statistically significant.

**Results:**

The mean value of TB staining was significantly higher in infertile group compared to normospermic group (p=0.005). Mean of sperm normal morphology was lower in idiopathic infertile men in comparison with normozoospermic men (p= 0.001). The highest negative correlation was obtained between sperm count and AB staining. Progressive motility was negatively correlated with AB and TB staining in both groups but there was no significant difference between AB staining and progressive motility in men normospermia group.

**Conclusion:**

Sperm chromatin staining using AB and TB showed a negative association between sperm chromatin condensation with sperm count, normal morphology and progressive motility. It seems that the AB and TB test may be useful for the assessment of male fertility potential.

## Introduction

Male factor has been implicated in about 50% of the infertile cases (1). An estimated 15% of men with normal basic sperm analysis have been associated with infertility (2, 3). Success rates of assisted reproductive technologies (ARTs) depend mostly on the structural and functional integrity of the gametes and have a decisive role. Current techniques employed in the field of andrology can be improved by the addition of a new tests for assessment of semen quality (4). It is evident from current studies that sperm chromatin defect is due to many reasons, which are associated with decreased fertilization rate and poor ART outcomes (5) and the higher incidence of pregnancy loss (6). Sperm chromatin carries also half of the genomic material to offspring and the integrity of sperm chromatin has fundamental importance for balanced transmission to future generations (7).

Subjectivity and variability of traditional semen parameters, abnormalities in the male genome, maybe disturbed chromatin packaging and sperm DNA are the conditions that disturb standard semen parameters (8–10). Inconsistently reports showed sperm chromatin screening was essential to infertility diagnosis (11). Normal and fertile donors were found to have lower levels of chromatin defects when compared to men receiving fertility services (12).

Sperm DNA integrity can be assayed in methods such as single cell electrophoresis or terminal deoxynucleotidyl transferase mediated dUTP nick end labeling (TUNEL) directly (13) and techniques such as sperm chromatin structural assay (SCSA) and chromomycine A3 (CMA3) indirectly. These methods necessitate the provision of expensive apparatus and they are often inaccessible to most andrology lab in IVF centers (14). Since abnormal chromatin integrity is more frequent in infertile men than fertile men, some techniques have been developed to evaluate sperm chromatin integrity status referred as cytochemical assays including aniline blue (AB) and toluidine blue (TB) staining tests (15–17).

These methods are based on the ability of some stains to assess the conformation of sperm chromatin, which in turn depends on DNA interaction with proteins (18, 19). The AB stain discriminates between lysine-rich histones and arginine/cysteine-rich protamines. This method provides a specific positive reaction for lysine and reveals differences in the basic nuclear protein composition of human spermatozoa (20, 21). TB is a basic nuclear dye used for metachromatic staining of the chromatin. The phosphate residues of sperm DNA in nuclei with loosely packed chromatin or impaired DNA become more prone to binding with basic TB dye, providing a metachromatic alteration due to dimerization of the dye molecules from light blue to purple–violet (22, 23). So far, these tests were introduced as simple, fast, and accurate for the analysis of sperm chromatin integrity. In addition, these methods do not require complex instrumentation (3). A significant difference and a wide range of chromatin defect were observed already between normal donors and patients with asthenozoospermia and oligoasthenozoospermia by using acridine orange (24). However, the correlation of AB and TB test with sperm count, motility, morphology and the assessment of sperm chromatin status are not well noted in different subfertile men.

With focusing on the chromatin integrity, evidence suggests a negative relationship between the incompetence of the chromatin material in sperm chromatin and the fertility potential of spermatozoa (25). Therefore, chromatin integrity can be measured as a reliable predictor of a couple’s ability to conceive. So, the objective of the present investigation was to evaluate semen samples for the status of sperm chromatin to find the relationship of the chromatin integrity with conventional sperm quality parameters in normozoospermia and different groups of subfertile men by using AB and TB tests.

## Methods

This study was approved by the Ethics Committee of Yazd Reproductive Sciences Institute, Shahid Sadoughi University of Medical Sciences, Yazd, Iran. In this retrospective study, the medical archives of 1386 couples referring to research and clinical center for infertility treatment between April 2011 to December 2016 were revised. Records of sperm from men who had undergone infertility treatment were reviewed. 342 men were normospermia whose partners were infertile and 1044 were infertile including idiopathic ones or the individuals affected by oligoasthenospermia, asthenospermia, and oligospermia. Patients with lack of sufficient clinical data and patients with testicular sperm extraction (TESE) or other surgical sperm retrieval were excluded from our study.

All specimens were collected by masturbation at the andrology laboratory, after an abstinence period of 2–3 days. After complete liquefaction at room temperature and before the semen preparation, the following characteristics were observed according to WHO guidelines: ejaculate volume, sperm concentration, total sperm count and motility (26). For each measurement, a 10 *μl* aliquot from the semen sample was loaded into a micro-cell chamber (Conception technologies, San Diego, CA) and analyzed for sperm concentration and motility. Seminal smears were stained with Diff-Quick stain and sperm morphology was assessed according to WHO criteria. 10 *μl* of the sample was taken on a slide and smear was prepared. Based on Diff-quik commercial kits Hemacolor® (Merck, Darmstadt, Germany) slides was kept for air drying and the slides were immersed in fixative (95% ethanol) for 15 seconds and the excess solution was drained by placing slides vertically on absorbent paper. The slides were immersed sequentially in step 1 to Eosin for 10 *s* and in step 2 in Methylene Blue for 5 *s*. Then the slides were rinsed briefly in tap water to remove excess stain and kept for air dry. Each slide was examined with oil immersion at ×1000 magnification in bright field microscopy (27).

### Aniline blue staining:

To perform this staining, fresh sperm smear of each case was air dried and then fixed in 3% buffered glutaraldehyde in 0.2 *M* phosphate buffer (pH=7.2) for 30 *min* at room temperature. Each smear was treated with 5% aqueous AB stain (BDH, Poole, UK, Cat. No. 34003) 5 *g* powder in 100 *ml* distilled water) in 4% acetic acid (pH=3.5) for 5 *min*. At least, 200 spermatozoa were counted in each slide by light microscopy (28). Unstained or pale blue stained cells and dark blue cells were considered normal and abnormal spermatozoa, respectively. At least 200 sperm cells were evaluated in each slide and the percentage of abnormal spermatozoa was reported.

### Toluidine blue staining:

To do this staining, after air drying of smears, they were fixed in fresh 96% ethanol-acetone (1:1) at 4°*C* for 30 *min* and then hydrolyzed in 0.1 NHCl at 4°*C* for 5 *min*. The slides were rinsed thrice in distilled water for 2 *min* and finally stained with 0.05% TB in 50% McIlvaine buffer (pH=3.5) for 10 *min* at room temperature (29). The chromatin quality of spermatozoa was determined according to metachromatic staining of sperm heads with the aid of light microscopy at ×1000 magnification. Pale blue sperm heads were considered as normal and dark blue or violet or purple spermatozoa were categorized in to abnormal cells.

### Statistical analysis:

Statistical analysis was performed using SPSS ver. 15.0 (SPSS Inc., Chicago. IL, USA). Regarding the analysis of semen parameters, a nonparametric test was used because of the large range of values. Statistical tests including non parametric Mann-Whitney U tests to determine the relationships between sperm parameters in two study groups, Kruskal-Wallis for multiple comparisons and spearman’s rank correlation for comparison of measured parameters were applied as well. Data is presented as mean± SD, and p<0.05 was considered statistically significant.

### Ethical approval:

Data collection and document review in this study were in accordance with the standards of the Ethics Committee of the Research and Clinical Center for Infertility, Shahid Sadoughi University of Medical Sciences, Yazd, Iran, and with the 1964 Helsinki declaration and its later amendments or comparable ethical standards.

## Results

Our data analysis showed that the mean age of the men in our study groups didn’t differ significantly, 37.8±4.2 in fertile patients vs. 35.1±5.6 in infertile patients (p=0.098). Mean of the ejaculation volume was 3.8±1.5 in normozoospermic men and 3.4±1.2 in infertile group and they were not significantly different (p=0.078). The semen parameters were analyzed in all the patients according to WHO criteria.

As listed in table 1, percentages of sperm count, normal morphology and progressive motility in idiopathic infertility are lower than normospermia but they are higher than subfertile groups. Also percentage of sperm with abnormal AB and TB staining was higher in idiopathic infertility compared to normospermia but it was higher than other infertile subgroups.

**Table 1.** Sperm parameters in normospermia group and infertile sub groups

**Sperm parameters**	**Normozoospermic n=342**	**Infertile n=1044**	**Idiopathic infertile ones n=754**	**Oligo spermia n=13**	**Asthenospermia n=184**	**Oligoasthenospermia n=93**	**p-value between groups**
**Count (×10^6^)**	94.33±48.22	73.55±54.96	88.41±54.22	10.00±3.10	50.09±34.66	8.34±4.20	0.001
**Motility (%)**							
**Progressive**	59.52±8.04	44.61±15.71	52.39±7.75	47.84±4.50	25.90±10.92	18.11±12.67	0.001
**Non progressive**	10.61±3.1	13.16±4.95	12.4±3.31	13.84±2.99	16.5±7.1	12.55±7.68	0.001
**Immotile**	29.59±5.91	42.4±17.39	35.57±11.63	39.07±5.58	56.99±14.28	69.35±19.13	0.001
**Morphology (%normal)**	39.17 ±9.24	16.76±10.75	18.64±11.00	14.69±9.60	3.63±8.30	8.01±6.65	0.001
**AB**	49.75±18.45	54.29±19.12	51.35±18.9	67.76±14.41	58.71±17.91	67.49±15.54	0.001
**TB**	58.95±20.78	62.52±20.06	60.95±21.00	69.76±12.63	64.17±17.9	71.00±13.79	0.001

Note: Values are presented by mean± SD. AB= Aniline blue staining, TB= Toluidine blue staining. Relationships between sperm parameters in normospermia and infertile groups were determined with the nonparametric Mann-Whitney U test. Also, P-value obtained from thedifference between means innormospermia and infertile sub groups was tested for significance, by Kruskal-wallis one way ANOVA on ranks and post hoc analysis was performed using Dunn’s for all pair- wise comparisons. p<0.05 was considered statistically significant

As shown in table 2, there is a negative correlation between sperm chromatin integrity with sperm count, normal morphology and progressive motility.

**Table 2.** Correlation between sperm parameters with sperm nuclear maturity tests

**Sperm parameters**	**Correlation coefficient**	**p**
**AB**		
Count (×10^6^)	−0.262 **	0.001
Motility (%)		
Progressive	−0.249**	0.001
Non progressive	0.078**	0.001
Immotile	0.258**	0.001
Morphology (%normal)	−0.237**	0.001
**TB**		
Count (×10^6^)	−0.147 **	0.001
Motility (%)		
Progressive	−0.249**	0.001
Non progressive	0.088**	0.001
Immotile	0.127**	0.001
Morphology (%normal)	−0.107**	0.001

Note. AB= Aniline blue staining, TB= Toluidine blue staining. Spearman’s nonparametric correlation coefficient was calculated for data that were not normally distributed.

** Correlation is significant at the 0.01 level (2-tailed)

Table 3 shows a negative correlation between sperm count and sperm with abnormal AB and TB staining in all groups but the correlation was significant just in idiopathic patients.

**Table 3.** Correlation between semen parameters and sperm chromatin quality tests based on study groups

**Sperm parameters**		**Normospermia**		**Infertile individuals**		**Idiopathic infertile individuals**		**Oligospermia**		**Asthenospermia**		**Oligoasthenospermia**	

		***AB***	**TB**	**AB**	**TB**	**AB**	**TB**	**AB**	**TB**	**AB**	**TB**	**AB**	**TB**
**Count (×10^6^)**	R	−0.095	−0.098	−0.244^**^	−0.144^**^	−0.157^**^	−0.092^*^	−0.061	−0.040	−0.031	−0.052	0.105	0.061
	p	0.080	0.070	0.001	0.001	0.001	0.011	0.842	0.896	0.673	0.486	0.317	0.561
**Progressive motility**	R	−.080	−0.118^*^	−0.248^**^	−0.132^**^	−0.141^**^	−0.069	−0.405	0.08	0.050	0.006	0.042	0.024
	p	0.139	0.029	0.001	0.057	0.001	0.057	0.170	0.086	0.500	0.941	0.687	0.816
**Non progressive motility**	R	0.025	0.113^*^	0.048	0.068	0.050	0.068	−0.099	−0.358	0–.046	0.017	−0.024	−0.103
	p	0.649	0.037	0.121	0.062	0.168	0.062	0.747	0.230	0.534	0.815	0.819	0.324
**Immotile motility**	R	0.084	0.065	0.213^**^	0.037	0.080^*^	0.037	0.361	0.149	0.032	0.023	−0.020	0.025
	p	0.121	0.233	0.001	0.313	0.029	0.313	0.225	0.627	0.665	0.761	0.850	0.808
**Morphology(% normal)**	R	−0.050	−0.027	−0.229^**^	−0.995	−0.132^**^	−0.995	−0.735^**^	−0.205	−0.273^**^	−0.095	−0.199	−0.050
	p	0.353	0.621	0.001	0.001	0.001	0.001	0.004	0.501	0.001	0.202	0.056	0.631

AB=Aniline blue staining, TB=Toluidine blue staining. Spearman’s nonparametric correlation coefficient was calculated for data that were not normally distributed.

*Correlation is significant at the 0.05 level (2-tailed), **Correlation is significant at the 0.01 level (2-tailed)

Also, a negative correlation exists between normal morphology and sperm with abnormal AB staining in all infertile groups and the correlation is significant except in oligoasthenozoospermia.

## Discussion

Semen analysis was done for normozoospermia and infertile groups and the results show a high significant difference (p=00.1) between two groups (Table 1). In total participants, chromatin results of AB and TB staining were correlated to sperm parameters (Table 1) and a negative correlation between sperm chromatin integrity and maturity with sperm count, normal morphology and progressive motility was observed (Table 2). These results are in line with Ali et al.’s study that recorded a high significant difference in sperm de-condensation between fertile and infertile men by AB and TB tests. Also, they revealed a high significant negative association between sperm AB^+^ and TB^+^ with sperm morphology, concentration and progressive motility (30). Although Hammadeh et al. reported a significant difference between patients and healthy donors by AB staining, they found no correlation between chromatin maturity with sperm morphology, count and motility (31). However, in another study, a significant association between chromatin condensation with strict morphology by AB and TB staining was found (22). Sellami et al. found a significant correlation between sperm chromatin maturity and the average number of sperm head abnormalities in infertile men by AB test but they didn’t find any correlation with sperm motility, vitality, and count (20). In another study, investigators reported a negative correlation between AB^+^ sperm cells with sperm morphology and progressive motility (32).

AB and TB staining results and sperm parameters were similar in normozoospermic men and idiopathic individuals. So, the etiology of chromatin defect should be sought in other factors like the aberration in protamine or apoptosis rather than the excess of histone or lower sperm condensation in male idiopathic infertility. In idiopathic cases, sperm parameters correlated with the chromatin results. Talebi et al. investigated the possible contribution of sperm in recurrent spontaneous abortion (RSA); they assessed semen quality of a group of male patients whose partners had at least three repeated abortions following natural conception. AB and TB staining results revealed that majority of samples in RSA patients exhibited high percentages of abnormal spermatozoa than cut off values regarding different cytochemical assays (33). Perhaps the difference between their report and ours is related to the fact that some participants who were classified as idiopathic were not typical cases of idiopathic infertility, for the reason that the current study is retrospective. Although the results of AB staining have shown a clear relationship between abnormal sperm chromatin condensation and male infertility (34), the correlation between the percentage of AB staining reacted spermatozoa and other sperm parameters remains controversial (35–37). Talebi et al. showed the results of analysis of sperm chromatin condensation and DNA integrity status using different assays between different experimental diabetic groups in mice. There were significant differences between groups regarding TB staining test, but there was not any significant difference in AB staining (38).

Most important is the finding that chromatin integrity as visualized by AB or TB staining is a predictor for ART outcomes, although these cannot determine the fertilization potential and the cleavage and pregnancy rates following ICSI (21, 39). In AB staining, basic proteins are loosely associated with DNA and unable to bind to the chromatin of normal sperm which is densely packed. A transition of histones to more basic protamines occurring during spermiogenesis neutralizes DNA charge and decreases the absorbing DNA-specific dyes (20, 22).

TB staining may be considered a quite reliable method to assess sperm chromatin. However, in normal sperm, the polymerization of the dye is stopped (40) and abnormal sperm may impair dye binding and polymerization. Looser chromatin has looser interactions with chromatin proteins, which can be easily displaced from the DNA in favor of metachromatic binding of the dye to DNA phosphate groups (41).

Abnormal nuclei as purple–violet sperm heads have been shown to be correlated with results by the acridine orange method. Moreover, correlations between the results of the TB staining, SCSA, and TUNEL tests have been verified (42).

AB staining cut-off at 6% normal morphology were 90% specificity and 62% sensitivity (28) and TB. TB staining threshold for the proportion of cells with violet heads was set at 45%, it provides 92% specificity and 42% sensitivity for male infertility detection (43).

## Conclusion

In the present study, a negative correlation existed between sperm chromatin integrity with sperm count, normal morphology and progressive motility by TB and AB staining, cut-off value in specificity and sensitivity of AB and TB staining was considered as a predictor for male infertility. So, the AB and TB test may be useful for the assessment of male fertility potential. Also, through focusing on the chromatin integrity, our data suggest a negative relationship between the incompetence of chromatin material and fertility potential of spermatozoa; chromatin integrity assessment by using AB and TB can be as a reliable predictor of conception in different subfertile groups.
